# Functional implication of heat shock protein 70/90 and tubulin in cold stress of *Dermacentor silvarum*

**DOI:** 10.1186/s13071-021-05056-y

**Published:** 2021-10-19

**Authors:** Desmond O. Agwunobi, Tongxuan Wang, Meng Zhang, Tianhong Wang, Qingying Jia, Miao Zhang, Xinyue Shi, Zhijun Yu, Jingze Liu

**Affiliations:** Hebei Key Laboratory of Animal Physiology, Biochemistry and Molecular Biology, College of Life Sciences, Hebei Normal University, Shijiazhuang, 050024 China

**Keywords:** Ixodidae, Cold tolerance, Heat shock protein, RNAi, Tubulin

## Abstract

**Background:**

The tick *Dermacentor silvarum* Olenev (Acari: Ixodidae) is a vital vector tick species mainly distributed in the north of China and overwinters in the unfed adult stage. The knowledge of the mechanism that underlies its molecular adaptation against cold is limited. In the present study, genes of *hsp70* and *hsp90* cDNA, named *Dshsp70* and *Dshsp90*, and tubulin were cloned and characterized from *D. silvarum*, and their functions in cold stress were further evaluated.

**Methods:**

The genome of the heat shock proteins and tubulin of *D. silvarum* were sequenced and analyzed using bioinformatics methods. Each group of 20 ticks were injected in triplicate with *Dshsp90*-, *Dshsp70*-, and tubulin-derived dsRNA, whereas the control group was injected with GFP dsRNA. Then, the total RNA was extracted and cDNA was synthesized and subjected to RT-qPCR. After the confirmation of knockdown, the ticks were incubated for 24 h and were exposed to − 20 °C lethal temperature (LT50), and then the mortality was calculated.

**Results:**

Results indicated that *Dshsp70* and *Dshsp90* contained an open reading frame of 345 and 2190 nucleotides that encoded 114 and 729 amino acid residues, respectively. The transcript *Dshsp70* showed 90% similarity with that identified from *Dermacentor variabilis*, whereas *Dshsp90* showed 85% similarity with that identified from *Ixodes scapularis*. Multiple sequence alignment indicates that the deduced amino acid sequences of *D. silvarum* Hsp90, Hsp70, and tubulin show very high sequence identity to their corresponding sequences in other species. Hsp90 and Hsp70 display highly conserved and signature amino acid sequences with well-conserved MEEVD motif at the C-terminal in Hsp90 and a variable C-terminal region with a V/IEEVD-motif in Hsp70 that bind to numerous co-chaperones. RNA interference revealed that the mortality of *D. silvarum* was significantly increased after injection of dsRNA of *Dshsp70* (*P* = 0.0298) and tubulin (*P* = 0.0448), whereas no significant increases were observed after the interference of *Dshsp90* (*P* = 0.0709).

**Conclusions:**

The above results suggested that *Dshsp70* and tubulin play an essential role in the low-temperature adaptation of ticks. The results of this study can contribute to the understanding of the survival and acclimatization of overwintering ticks.

**Graphical abstract:**

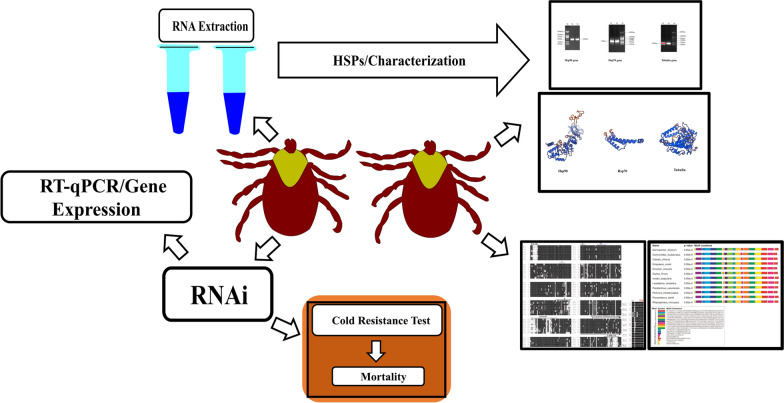

**Supplementary Information:**

The online version contains supplementary material available at 10.1186/s13071-021-05056-y.

## Background

Ticks are important zoonotic pathogen vectors with global distribution, ranking second only to mosquitoes as arthropod vectors threatening human and animal life [1, 2]. They are non-permanent obligate blood-feeding ectoparasites that spend most of their lifetime off-host, which are expected to be most affected by the off-host abiotic conditions [3, 4]. In temperate regions, the low winter temperature plays a key role in influencing the survival, development, occurrence, and expansion of ticks [5–7].

*Dermacentor silvarum* tick is a known vector of an infectious agent, *Rickettsia sibirica*, that causes the condition known as North Asian tick typhus and has also been implicated as the vector of two pathogens (*Theileria equi* and *B. caballi*) that cause theileriosis and babesiosis [2]. In China, *D. silvarum* tick has been implicated as a reservoir that maintains the tick-borne encephalitis virus via transstadial and transovarial transmission [8]. Other pathogenic agents vectored by *D. silvarum* include *Rickettsia raoultii, R. heilongjiangensis, R. slovaca* (collectively known as spotted fever group rickettsiae), and the causative agent of human monocyclic *Ehrlichia* (*Ehrlichia chaffeensis*) [9, 10].

The distribution of *D. silvarum* ranges from Russia to Mongolia and the north of China [11]. In the north of China, the adult and nymphal *D. silvarum* were found mostly on the ears of sheep, whereas the larvae were found mainly on the vegetation [12]. Adult population was dominant from late February to late May (peaked in mid-April); the nymphs were widespread from late June to late September (peaked in mid-August) when they struggle with occasional low temperatures, while the larvae were distributed from early June to early September (peaked in mid-July) [2, 12]. During the low-temperature months after October, an overwintering male adult population was found on sheep, whereas the vegetation was void of free-living adults [12].

Arthropods, including ticks, can be susceptible to stress caused by fluctuations in environmental temperature, and specific protective mechanisms have to be elicited within their system for them to survive. Heat shock proteins (Hsps) are one of the classes of protective agents that contribute immensely to the overwintering cold tolerance of arthropods [13]. They function mainly as molecular chaperones that play a significant role in preserving enzymatic functions and other essential proteins [14] and participate in the folding and conformational regulation of proteins [15]. Hsp90 and Hsp70 are highly conserved ATP-dependent molecular chaperones that are functionally associated with protein homeostasis [16, 17]. They are the two major cytosolic chaperones whose degradation and folding characteristics are vital to the maintenance of native proteins and prevention of denatured protein aggregation [15, 18]. Their participation in the remodeling of a variety of client proteins and involvement in numerous cellular functions including signal transduction, protein trafficking, and receptor maturation underscore their indispensable roles in eukaryotes [16, 19].

In most organisms, Hsp70s and Hsp90s have been documented to upregulate during environmental stress [20–23]. Hsp70 and Hsp90 families of molecular chaperones play a vital role in the maintenance of protein homeostasis and cellular recovery as a result of environmental stresses including heat and oxidative stress [24]. As two of the most prominent Hsps and together with Hop (Hsp70–Hsp90 organizing protein), which acts as a link between the two chaperones, they form a functionally active complex in eukaryotes [24, 25]. A strong collaboration exists between Hsp70 and Hsp90 in that they undergo large conformational changes with their adaptor molecule (Hop), which enhances the folding, stabilization, and assembly of clients (misfolded, structurally unstable, and mutated proteins) [26–28]. Although it is known that Hsp70 facilitates client delivery to Hsp90, both can equally interact with other chaperones/co-chaperones in a coordinated manner to bring about regulation, function, and stability [27]. For instance, glucocorticoid receptor (GR) folding and the assembly of GR-Hsp90 heterocomplexes are mediated by the coordinated activities of Hsp90, Hsp70, Hop, Hsp40, and p23 [27, 29]. While Hsp70 inactivates GR via partial unfolding/ligand release, Hsp90 reverses this inactivation via refolding/ligand binding, and the ligand-binding recovery requires ATP hydrolysis on Hsp90, the Hop, and p23 co-chaperones [27]. Additionally, the client transfer from Hsp70 seems to be regulated by Hsp90 ATP hydrolysis via coupling of the ATP cycles of the two chaperones, which is generated by their contacts within the GR:Hsp70:Hsp90:Hop complex as shown by cryoelectron microscopy image [27]. Meanwhile, it is noteworthy that the C-terminal domains of Hsp90 and Hsp70 comprise highly conserved sequence EEVD-COOH, which interacts with the tetratricopeptide repeat (TPR) domain of co-chaperones [30, 31].

The level of Hsp expression fluctuates to ensure adaptation of the organism to different stresses, which include variable temperatures and different physiological stages, some chemicals and drugs, gamma radiation, ultraviolet light, bacterial and viral infection, among others [32–34]. Several tick species go through diapause in response to different stresses such as cold, heat, and hunger, and Hsps actively participate in the physiological response to stress in insects and ticks during diapause [13, 22]. Tubulins are heterodimeric proteins that assemble in a head-to-tail arrangement to form a linear protofilament, and they are known to be the basic building block of microtubules [35]. Microtubules putatively enhance cold tolerance given that some microtubules (the non-cold adapted types) are depolymerized and disassembled during cold temperatures [36]. The cytoskeleton of eukaryotic cells is composed of microtubules involved in many vital processes such as cell division, intracellular transport, and ciliary and flagella-driven motility [35].

RNA interference (RNAi) is a gene-silencing or gene-knockdown mechanism present in ticks and several eukaryotes, which involves the activation of a sequence-specific degradation of cognate mRNAs by double-stranded RNA (dsRNA) [37]. As a well-conserved post-transcriptional mechanism, it has been an effective means to study the function of tick genes [38–40]. In the present study, *D. silvarum hsp70* (*Dshsp90*), *hsp90* (*Dshsp90*), and tubulin genes were cloned and characterized, and their functions during cold stress were evaluated using RNAi in the hope of expanding our knowledge on the molecular mechanism underlying the cold response of ticks.

## Methods

### Tick rearing and acclimation

Adult ticks were collected from Xiaowutai National Nature Reserve, Zhangjiakou (114°47ʹ–115°28ʹE, 39°50ʹ–40°6ʹN), in Hebei Province, China. The unfed ticks were raised in an environmental chamber (Beijing Oriental Science and Technology Development Co., Ltd., Beijing, China) with the temperature set at 26 °C, a relative humidity of 90%, and light and dark regime of 12 h each [41]. The ticks were morphologically identified according to Teng and Jiang [11]. The ticks were fed on the ears of domestic rabbits (*Oryctolagus cuniculus* L.). Institutional ethical and animal care guidelines were followed, and all protocols were performed in accordance with the China Guide for the Care and Use of Laboratory Animals (protocol number: IACUC-157031).

### RNA extraction and synthesis of the first-strand cDNA

Total RNA was extracted from the ticks according to the manufacturer's protocol using TransZol Up Plus RNA Kit (TransGen Biotech Co., Ltd., Beijing, China). RNA quantification was carried out using Nano Drop® ND-1000 Spectrophotometer (Thermo Fisher Scientific, Waltham, MA, USA). The RNA integrity was evaluated by agarose gel electrophoresis, and RNA samples were stored at − 80 °C until use. First-strand cDNA was synthesized from 1 μg total RNA using TransScript® One-Step gDNA Removal and cDNA Synthesis SuperMix (TransGen Biotech Co., Ltd, Beijing, China) and an oligo-dT primer. PCR conditions were 35 cycles of 30 s at 94 °C, 30 s at the *T*_m_ of the gene-specific primers, and 30 s at 72 °C, preceded by an initial 2 min denaturation at 95 °C, and then a final extension at 72 °C for 10 min carried out on an Applied Biosystems® Veriti® 96-Well Thermal Cycler (Life Technologies, Ltd., Marsiling, Singapore). The quality of PCR amplicons was checked using 1% agarose gel. Bands with expected sizes were excised and purified by a gel extraction kit (CWBIO, Beijing, China). All the primers were designed with DNAMAN (Lynnon Biosoft, San Ramon, CA, USA) and Primer Premier version 5.0 for Windows (Premier Biosoft International, Palo Alto, CA, USA) (Additional file 4: Table S1). Information from the GenBank sequences and transcriptomic data [42] was used to design the primers for target genes in *D. silvarum*.

### Bioinformatic analysis for the *Dshsp90*, *Dshsp70*, and tubulin gene

The gel strips of the purified PCR products obtained after electrophoresis were sent to Invitrogen (Beijing, China) for sequencing (Additional file 1: Figure S1) and then analyzed by BLASTn (http://www.ncbi.nlm.nih.gov/BLAST) search in the National Center of Bioinformatics Information (NCBI) GenBank. The complete genome sequence of the heat shock proteins and tubulin of *D. silvarum* were deposited in the NCBI nucleotide database under accession numbers MT646143 (Dshsp90), MT679220 (Dshsp70), and MT646144 (tubulin gene). The open reading frames (ORF) of *Dshsp90, Dshsp70*, and tubulin were identified by NCBI ORF finder (https://www.ncbi.nlm.nih.gov/orffinder/). To compare the multiple sequence alignment of *D. silvarum* Hsp90/Hsp70/tubulin in other species, BLASTp in GenBank (http://www.ncbi.nlm.nih.gov/), Clustal W2 (http://www.ebi.ac.uk/Tools/clustalw2/index.html), and BOXSHADE 3.21 (http://www.ch.embnet.org/software/BOX_form.html) were used. Multiple Em for Motif Elicitation (MEME) program version 5.1.1 (http://meme-suite.org/tools/meme) was employed to compare conserved motifs between *D. silvarum* Hsp90/Hsp70/tubulin and their homologs from other species [43]. Swiss-Model (http://swissmodel.expasy.org/) was employed for the protein tertiary structure prediction.

### Synthesis of dsRNA

Oligonucleotide primers comprising T7 promoter sequences at the 5ʹ end for in vitro transcription and synthesis of dsRNA were used to amplify cDNA encoding *Dshsp90*, *Dshsp70*, and tubulin. All the oligonucleotide primers used for this study were synthesized by Invitrogen™ (Beijing, China) (Additional file 4: Table S1). The region of each transcript's nucleic acid sequence targeted for silencing was randomly selected, and the base pair sizes ranged from 120 to 635. Purification of T7-incorporated PCR products was carried out using the High Pure PCR Cleanup Micro Kit™ (Roche Diagnostics, Mannheim, Germany). The purified amplicon was used as templates to produce dsRNA using the T7 Ribomax Express RNAi System (Promega, Madison, WI, USA).

### Injection of ticks with dsRNA

The procedure for the injection of *D. silvarum* female ticks was performed, as previously described by Kocan et al. [44] and Agwunobi et al. [45]. Two microliters of dsRNA solutions of *Dshsp90*, *Dshsp70*, and tubulin was injected into the hemocoel of unfed female ticks through the third and fourth coxa at 1 μg/tick concentration using a 10-µl Microliter™ Syringes (Hamilton, NV, USA). A group of 20 ticks were injected in triplicate with dsRNA. The control group was injected with synthesized GFP dsRNA. After the injection, the ticks were placed in the environmental incubator at 26 ºC for 24 h (12 h light/12 h dark).

Thereafter, total RNA was extracted from groups of ten ticks from each dsRNA-treated group, and their cDNA was synthesized as stated above. The cDNA was subjected to RT-qPCR with an Mx3005P qPCR system (Agilent Technologies, Santa Clara, CA, USA) using TransStart® Top Green qPCR SuperMix (TransGen Biotech) according to the manufacturer’s instructions, using *Hsp* and tubulin gene-specific primers, such as *Hsp90* forward and reverse primers (RT-qPCR), *Hsp70* forward and reverse primers (RT-qPCR), and tubulin forward and reverse primers (RT-qPCR) (Additional file 4: Table S1). The RT-qPCR cycle profile was as follows: 94 ºC for 30 s, then 40 cycles of denaturation at 94 °C for 5 s, annealing at 60 °C for 30 s, and extension at 94 °C for 2 min. Triplicate reactions for each DNA sample were contained in each plate. After each assay, melting curves were traced to confirm that the fluorescence signal was retrieved from specific PCR products and also to ensure the absence of primer dimers. Ct represents the relative gene expression levels for each gene in each sample, and it was transformed into relative values, or it was relatively quantified (RQ) by the 2^−ΔΔCT^ method, where ΔΔC_T_ = (C_T, Target_−C_T, Actin_) _sample_–(C_T, Target_−C_T, Actin_) _control_ [46]. The PCR products were run in 1.5% TAE (Tris-acetate-EDTA) agarose gel and stained in a TAE buffer with ethidium bromide. *Actin* was used as the loading control.

### Cold resistance test

After the confirmation of knockdown, the ticks were incubated for 24 h (26 ± 1 °C, 85 ± 5% relative humidity, and a 16 h light/8 h darkness photoperiod). Then, the ticks were exposed to − 20 ºC lethal temperature (LT50) [47] in a 2-h incubation period in a low-temperature thermostatic water bath (Meixiang Instrument Co., Ltd., Shanghai, China). The sublethal temperature of − 20 °C was chosen given that the discriminating temperatures (resulting in 20% survival) for the females and males were 21.7 and − 22.6 °C, respectively [47]. The ticks were then removed from the cold water bath, and the mortality was calculated. Each experimental unit comprised 20 ticks treated in triplicate (60 ticks for each treatment).

### Statistical analysis

One-way ANOVA test was used to compare the differences among different treatment groups, and Tukey’s test was used for post hoc analysis. *P* < 0.05 was considered statistically significant. The data were analyzed using Graph Prism, version 8.0.2, for Windows (GraphPad Software, Inc., San Diego, CA, USA).

## Results

### Target gene cloning results

The total RNA of the female tick *D. silvarum* was extracted, reverse transcribed into cDNA, and PCR-amplified using the cDNA as a template. The resulting PCR product was subjected to agarose gel electrophoresis. The target fragments of the *Dshsp90*, *Dshsp70*, and tubulin genes were 408 bp, 280 bp, and 192 bp, respectively (Additional file 2: Figure S2).

### Bioinformatics and motif analysis

**Fig. 1:**
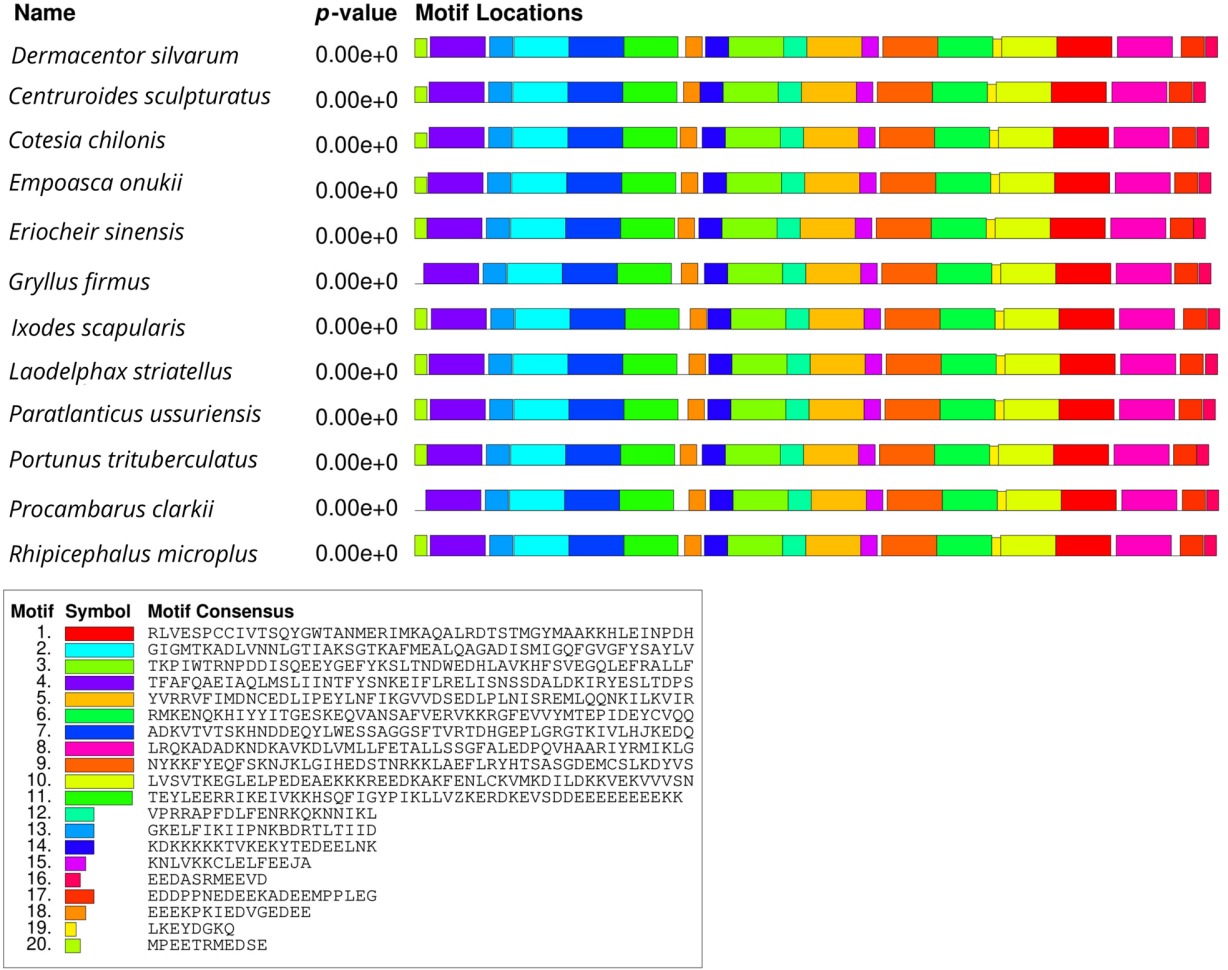
Analysis of conserved motifs present in *Dermacentor silvarum* heat shock protein 90 (Hsp90) compared with their homologs. Each colored box represents a motif in the protein, with the name indicated in the box at the bottom

**Fig. 2:**
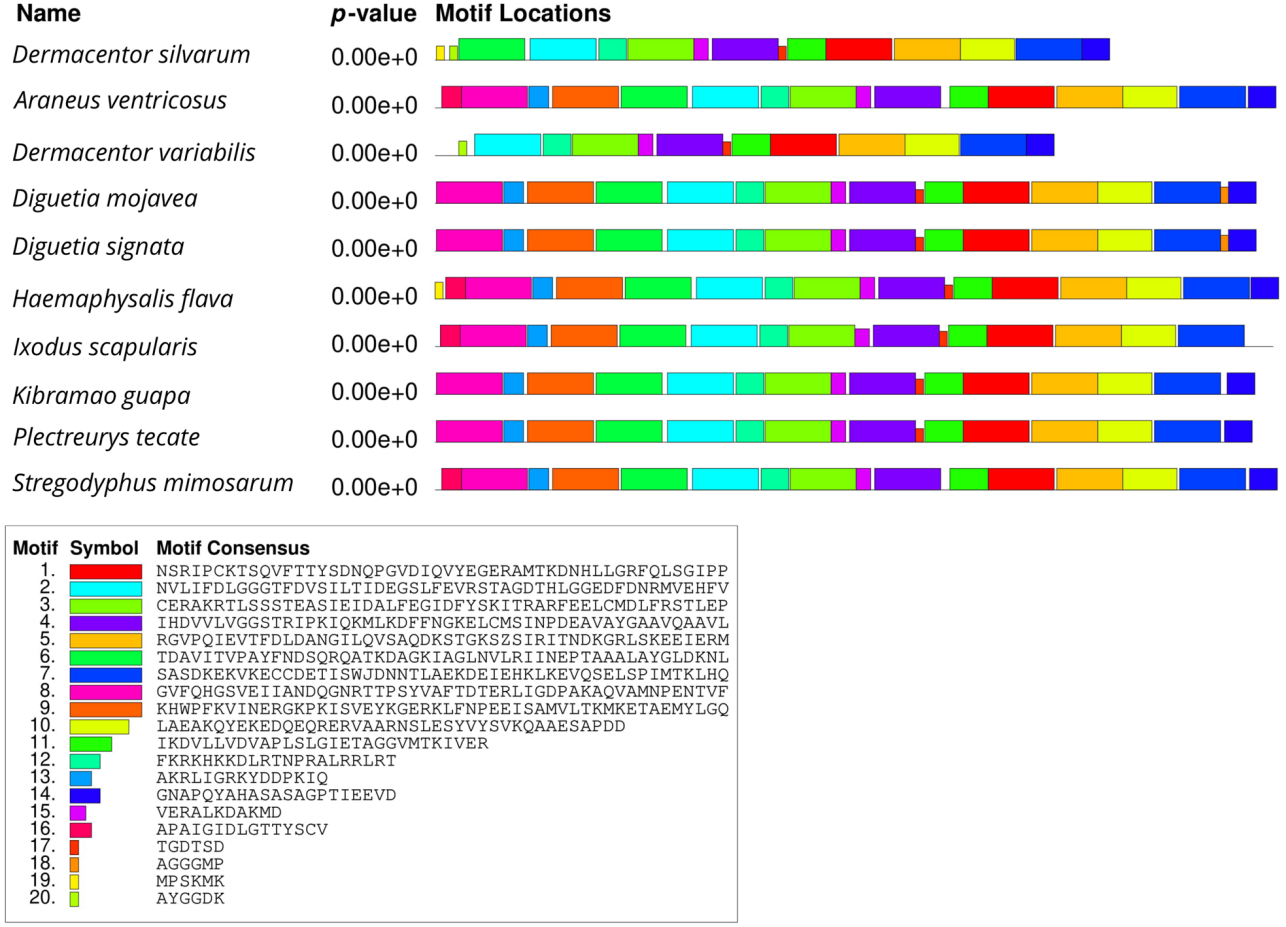
Analysis of conserved motifs present in *Dermacentor silvarum* heat shock protein 70 (Hsp70) compared with their homologs. Each colored box represents a motif in the protein, with the name indicated in the box at the bottom

**Fig. 3:**
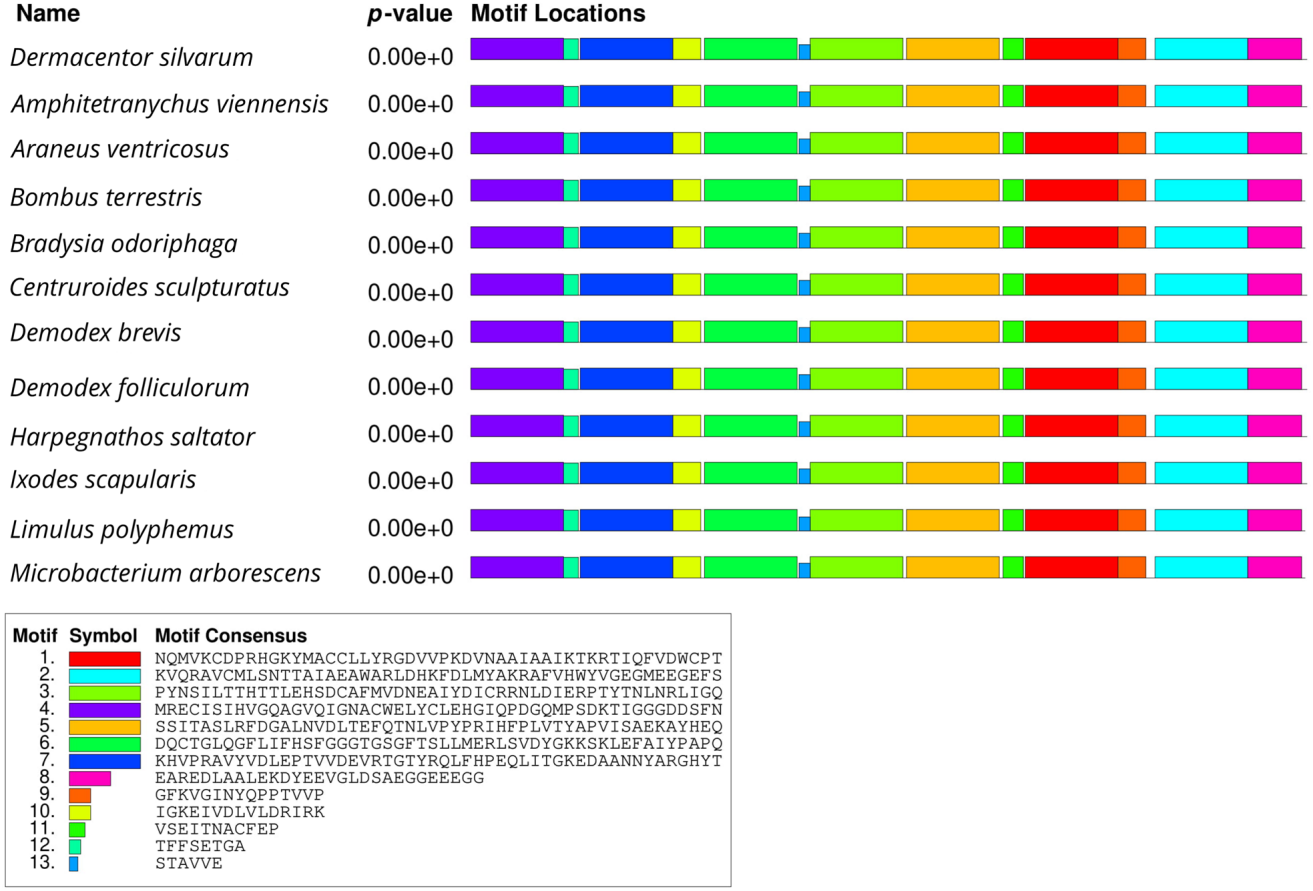
Analysis of conserved motifs present in *Dermacentor silvarum* tubulin protein compared with their homologs. Each colored box represents a motif in the protein, with the name indicated in the box at the bottom

The open reading frames (ORF) of *Dshsp90, Dshsp70*, and tubulin were obtained from ORFfinder in NCBI. The ORF of the *Dshsp90* gene was 2190 nucleotides (nt) in length (187 bp to 2376 bp) and encodes 729 amino acids, whereas that of the *Dshsp70* gene was 345 nt in length (2 bp to 347 bp) and encodes 114 amino acids. The tubulin gene ORF has a length of 1356 nt ranging from 102 to 1457 bp and encodes 451 amino acids. Motif analysis of Hsp90, Hsp70, and tubulin protein of *D. silvarum* was carried out to determine patterns of the conserved motifs and sequences by MEME. Multiple alignments with the homologs of *D. silvarum* Hsp90 amino acid sequence revealed 20 consensus motifs indicating greater identity with 11 species of arthropods (Fig. 1). Similarly, the Hsp70 amino acid sequence of *D. silvarum* showed 20 consensus motifs with nine arthropods (Fig. 2), whereas tubulin amino acid sequence multiple alignments showed 13 consensus motifs with 11 species of arthropods (Fig. 3).

### Sequence analysis of Hsp90, Hsp70, and tubulin

**Fig. 4:**
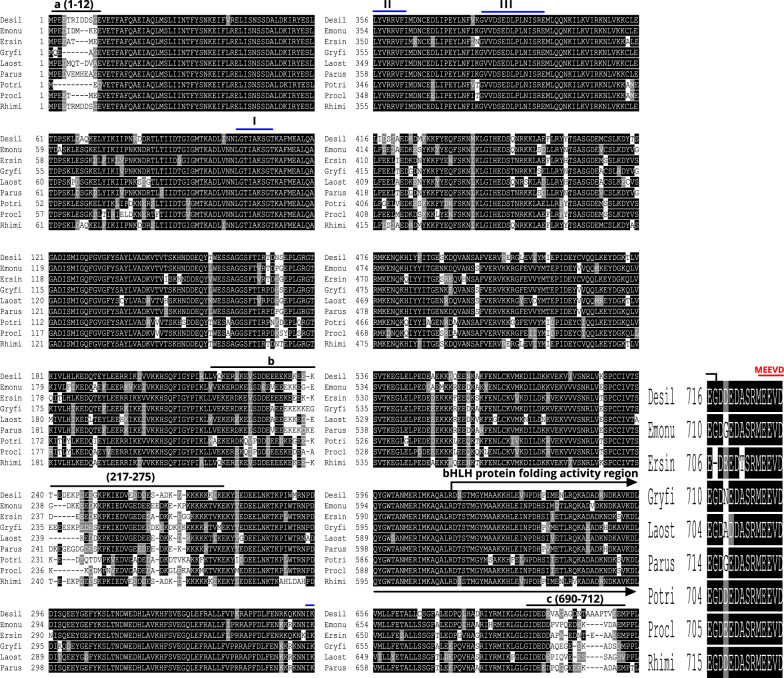
Sequence alignments of Hsp90 amino acid sequences indicate high similarity to their counterparts in other species. The sequence of Desil-Hsp90 is compared with Hsp90s from *Empoasca onukii* (Emonu), *Eriocheir sinensis* (Ersin), *Gryllus firmus* (Gryfi), *Laodelphax striatellus* (Laost), *Paratlanticus ussuriensis* (Parus), *Portunus trituberculatus* (Potri), *Procambarus clarkia* (Procl), and *Rhipicephalus microplus* (Rhimi). Three variable regions, **a** (aa 1–12), **b** (aa 217–275), and **c** (aa 690–712), are highlighted; a bHLH protein folding activity region is bracketed

The primary amino acid sequence of the heat shock protein of *D. silvarum* (Desil-Hsp90) constitutes several characteristics diagnostics of protein families of Hsp90. Desil-Hsp90 can be delineated into three domains as shown in Fig. 4, which includes a conserved N-terminal domain (aa 1-215) that is an ATP- or geldanamycin-binding sequence, a middle domain (aa 275-612), and a dimerization domain at the C-terminal (aa 613-717) that has a conserved bHLH protein folding activity region as denoted by the arrow-bracket in Fig. 4. Three perfect signatures of Hsp90 proteins that are well-conserved sequences were identified (Fig. 4), which include LGTIAKSGT, IKLYVRRVFI, and GVVDSEDLPLNISRE [48, 49]. The core sequence, which is the pentapeptide MEEVD found at the end of C-terminus (Fig. 4) that binds to the tetratricopeptide repeats (TPRs) domain of Hsp90 co-chaperones [50], indicates that Hsp90 of *D. silvarum* is a cytosolic Hsp protein [48]. Finally, there were three variable regions in Desil-Hsp90 (Fig. 4), which include an N-terminal region (aa 1–12), a linker region (aa 217–275), and a C-terminal domain region (aa 690–712) upstream of the MEEVD motif.

**Fig. 5:**
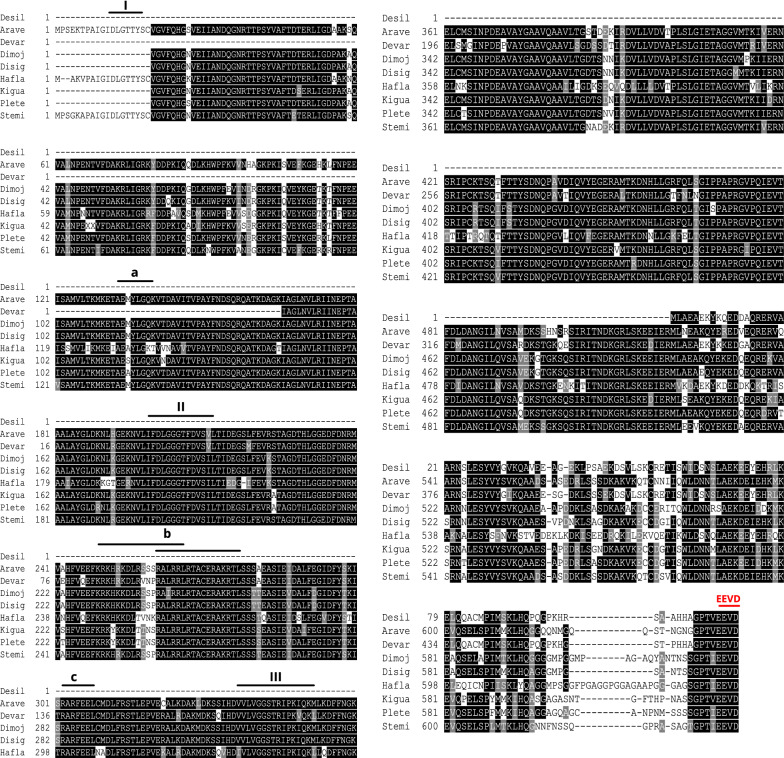
Sequence alignments of Hsp70 amino acid sequences indicate high similarity to their counterparts in other species. The sequence of Desil-Hsp70 is compared with Hsp70s from *Araneus ventricosus* (Arave), *Dermacentor variabilis* (Devar), *Diguetia mojavea* (Dimoj), *Diguetia signata* (Disig), *Haemaphysalis flava* (Hafla), *Kibramoa guapa* (Kigua), *Plectreurys tecate* (Plete), and *Stegodyphus mimosarum* (Stemi). A deduced ATP-GTP binding site, a putative bipartite nuclear localization signal, and a non-organellar consensus motif are highlighted as **a**, **b**, and **c**, respectively

**Fig. 6:**
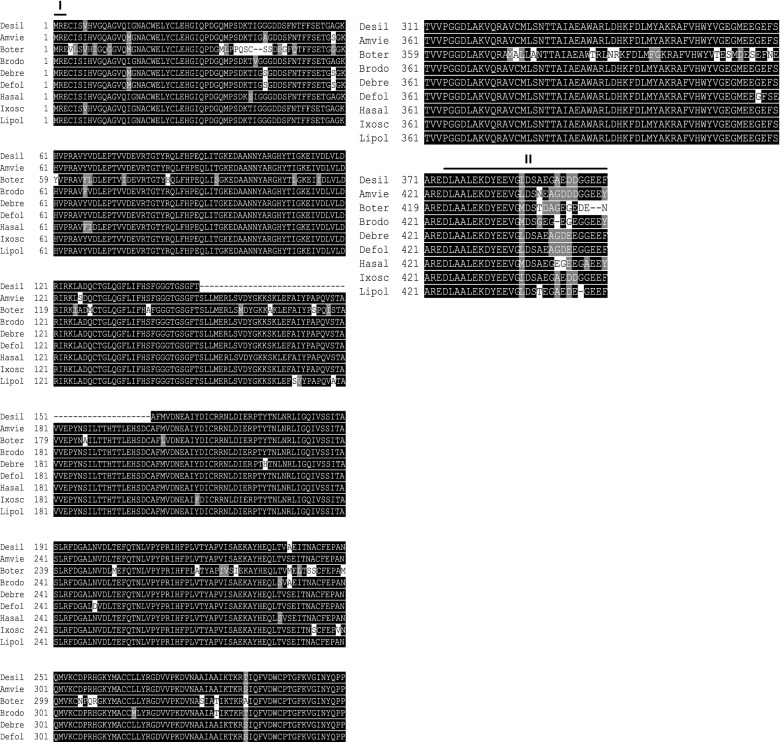
Sequence alignments of tubulin amino acid sequences indicate high similarity to their counterparts in other species. The sequence of Desil-tubulin is compared with tubulins from *Amphitetranychus viennensis* (Amvie), *Bombus terrestris* (Boter), *Bradysia odoriphaga* (Brodo), *Demodex brevis* (Debre), *Demodex folliculorum* (Defol), *Harpegnathos saltator* (Hasal) *Ixodes scapularis* (Ixosc), and *Limulus polyphemus* (Lipol). Two typical sequences at the N-terminal and C-terminal were highlighted

Although the deduced Desil-Hsp70 amino acid sequence was comparatively short, it strongly aligned with the Hsp70 of other species exhibiting some distinctive features of the Hsp70 family. These features include a C-terminal variable domain that ends with a conserved EEVD motif and three signature sequences labeled in Fig. 5 as I, II, and III, and they include IDLGTTYS, IFDLGGGTFDVSIL, and VVLVGGSTRIPKIQN [49, 51]. Other distinct features were three characteristic motifs (Fig. 5), which include (i) AEAYLGTS (labeled as a), which is a deduced ATP-GTP binding site [49, 51]; (ii) KRKFKKDIKNNSRALRRL (labeled as b), which is a deduced bipartite nuclear localization signal [49, 51]; and (iii) RARFEEL (labeled as c), which is referred to as a non-organellar consensus motif [49, 52]. For the Desil-tubulin amino acid sequence (Fig. 6), two typical sequences were identified of which one was at the beginning of the N-terminal (MRE labeled as I) [53] and the other was at the end of the C-terminal (DLAALEKDYEEVGIDSAEGAEDDGGEEF labelled as II) [54].

Multiple sequence alignments indicated that the deduced amino acid sequences of *D. silvarum* Hsp90, Hsp70, and tubulin showed very high sequence identity to their corresponding sequences in other species, including *Emposca onukii*, *Eriocheir sinensis*, *Gryllus firmus*, *Laodelphax striatellus*, *Paratlanticus ussuriensis*, *Portunus trituberculatus*, *Procambarus clarkia*, *Rhipicephalus microplus*, *Araneus ventricosus*, *Dermacentor variabilis*, *Diguetia mojavea*, *Diguetia signata*, *Haemaphysalis flava*, *Kibramoa guapa*, *Plectreurys tecate*, *Stegodyphus mimosarum*, *Amphitetranychus viennensis*, *Bombus terrestris*, *Bradysia odoriphaga*, *Demodex brevis*, *Demodex folliculorum*, *Harpegnathos saltator*, *Ixodes scapularis*, and *Limulus polyphemus* as shown in Fig. 5a–c. Desil-Hsp90 exhibited an average of 97% identity to Emonu-Hsp90 (GenBank#AIM18803.1), Ersin-Hsp90 (GenBank# ADE60732.1), Gryfi-Hsp90 (GenBank# ADK64952.1), Laost-Hsp90 (GenBank# AJK31518.1), Parus-Hsp90 (GenBank# AFP54306.1), Potri-Hsp90 (GenBank# ACQ90226.1), Procl-Hsp90 (GenBank# AGB14568.1), and Rhimi-Hsp90 (GenBank# AYQ96077.1), while Desil-Hsp70 showed an average of 98% identity to Arave-Hsp70 (GenBank# GBL92420.1), Devar-Hsp70 (GenBank# ABG47444.1), Dimoj-Hsp70 (GenBank# ABQ12805.1), Disig-Hsp70 (GenBank# ABQ12808.1), Hafla-Hsp70 (GenBank# AIS39468.1), Kigua-Hsp70 (GenBank# ABQ12821.1), Plete-Hsp70 (GenBank# ABQ12825.1), and Stemi-Hsp70 (GenBank# KFM69762.1). Desil-tubulin exhibited an average of 99% identity to Amvie-tubulin (GenBank# QGZ07430.1), Boter-tubulin (GenBank# XP_012172637.1), Brodo-tubulin (GenBank# ANA52573.1), Debre-tubulin (GenBank# QGQ62238.1), Defol-tubulin (GenBank# QGQ62236.1), Hasal-tubulin (GenBank# XP_011151658.1), Ixosc-tubulin (GenBank# EEC05915.1), and Lipol-Hsp70 (GenBank# XP_013790668.1).

Additionally, the sequence homology alignment of the three genes conducted via NCBI blast showed that *Dshsp90* gene and the *Ixodes scapularis Hsp90* gene have a similarity of 85%, whereas *Dshsp70* gene and *D. variabilis Hsp70* gene have a similarity of 90%. The *D. silvarum* tubulin gene and *I. scapularis* alpha-tubulin gene have a similarity of 85%.

### Protein secondary structure

After the translation of the three genes to proteins, the proportion of α-helix, β-sheet, and random coil in the sequences was analyzed for the protein secondary structure prediction. The results showed that α-helix, β-sheet, and random coil accounted for 43.76%, 15.09%, and 41.15%, respectively, in Hsp90. In Hsp70, α-helix, β-sheet, and random coil accounted for 71.93%, 0%, and 28.07%, respectively, whereas α-helix, β-sheet, and random coil accounted for 34.66%, 17.71%, and 47.63% in tubulin, respectively (Table 1). The results of the protein secondary structure show that the three peptide chains have a large proportion of α-helix and random coil structure, which may indicate more stability of the protein conformation.

**Table 1:** Secondary structure prediction results showing the percentage of α-helix, β-sheet, and random coil in Hsp90, Hsp70, and tubulin

Protein	α-helix	β-sheet	Random coil
Hsp90	43.76	15.09	41.15
Hsp70	71.93	0	28.07
Tubulin	34.66	17.71	47.63

### Protein tertiary structure prediction

Hsp90 protein is composed of a large number of α-helix and random coil structures, with only a small amount of β-sheet structure. Hsp90 protein contains two polar regions (high-polar A and B regions), and also seven lysines were found in the middle of the molecule. In the z-region, the polar amino acids in the A and B regions were located on the surface of the protein, mainly forming an α-helix, which may participate in the interaction between the proteins.

**Fig. 7:**
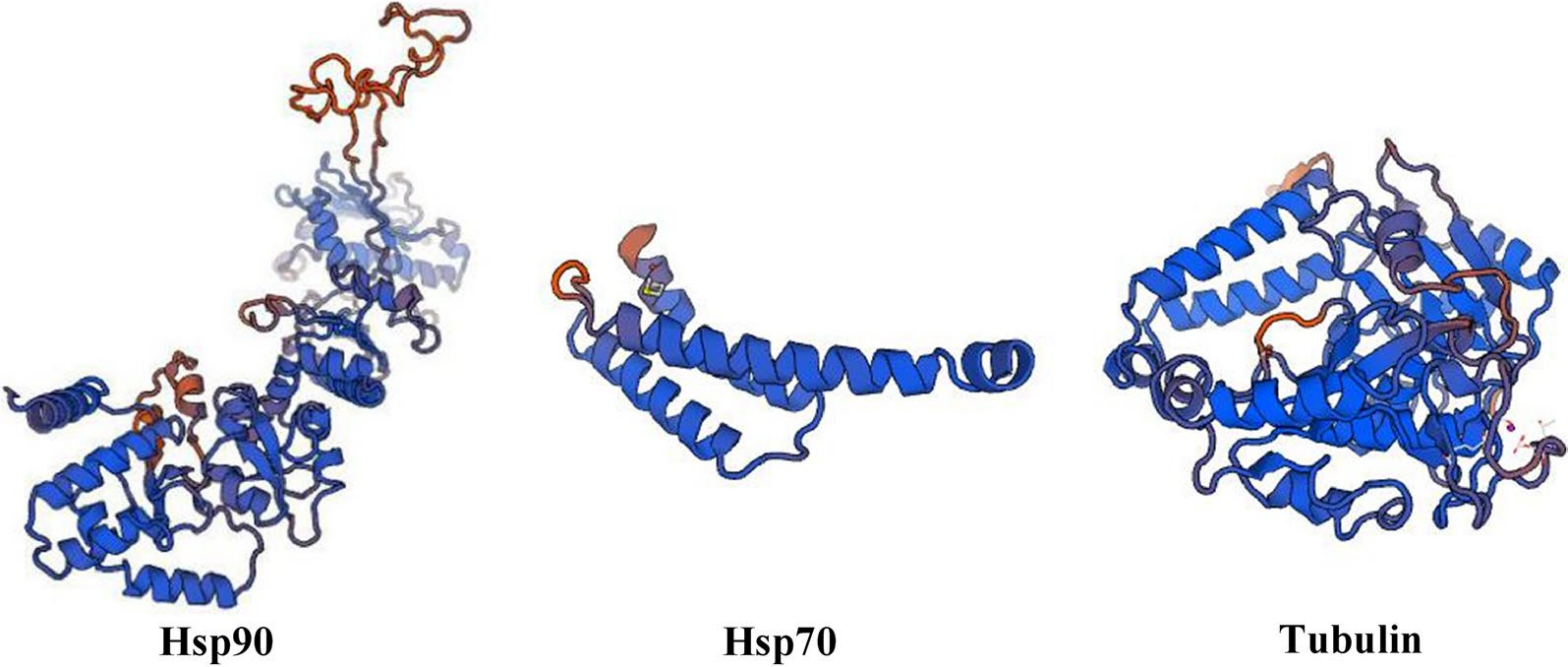
Protein tertiary structure prediction. **a** Hsp90, confidence score = 0.72 and 81.31% coverage; **b** Hsp70, confidence score = 0.53 and 59.14% coverage; **c** tubulin, confidence score = 0.84 and 95.51% coverage. The three peptide chains all have a large proportion of α-helix, which may indicate more stability of the protein conformation

Hsp70 is composed of an α-helix and random coil structure, which mainly acts as a molecular chaperone between cells. Tubulin was centered on two β sheets, surrounded by multiple α-helices, and has a super secondary structure composed of βαβ at the carbon end. It is speculated that it may be a microtubule-binding protein and motor protein binding domain (Fig. 7). The reliability range of the global model quality estimation (GMQE) is from 0 to 1. The GMQE values of Hsp90, Hsp70, and tubulin were 0.72 with 81.31% coverage, 0.53 with 59.14% coverage, and 0.84 with 95.51% coverage, respectively. Thus, the tertiary structure prediction models of Hsp90, Hsp70, and tubulin generally indicated higher confidence.

### Gene cloning results after T7 promoter ligation

Gene cloning was performed after the attachment of the T7 promoter recognition sites on both forward and reverse end, and the results of *Dshsp90*, *Dshsp70*, and tubulin gene bands (446 bp, 318 bp, and 230 bp, respectively) were all clear and correct (Additional file 3: Figure S3).

### Gene expression after RNAi

**Fig. 8:**
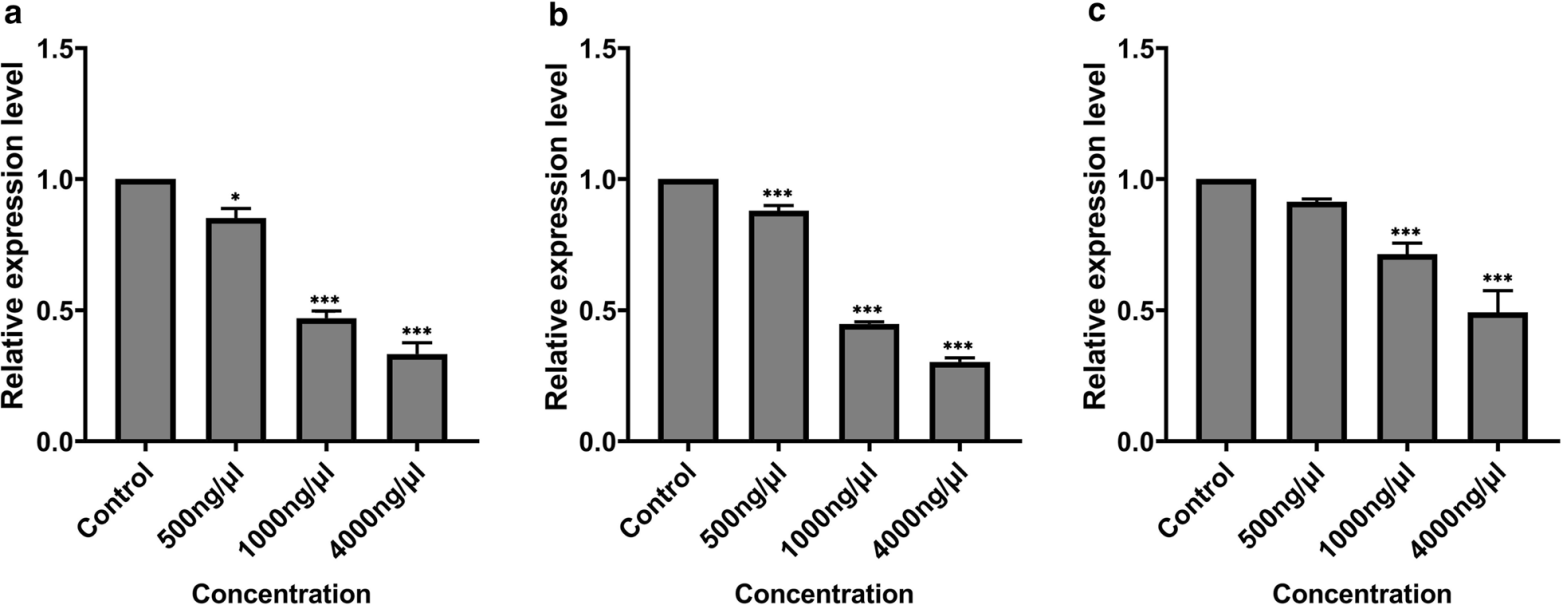
Gene expression results after RNAi. **a** *Dshsp90* gene; **b** *Dshsp70* gene; **c** tubulin gene. Data were presented as the means (± standard error, SE). Statistical analysis was calculated at a 5% probability using a one-way ANOVA followed by Turkey's test. Asterisks above bars indicate a significant difference between each treatment group and the GFP-dsRNA injected group (control), *P < 0.05, ***P < 0.001

Interference injection was performed with 2 μl of 500 ng/μl, 1000 ng/μl, and 4000 ng/μl dsRNA. The total RNA was extracted, and the expression was detected (Fig. 8). At the concentrations of 500 ng/μl, 1000 ng/μl, and 4000 ng/μl, the expression of *Dshsp90* gene was reduced by 15%, 53%, and 67%, respectively, compared with the control. The *Dshsp70* gene was downregulated by 12%, 55%, and 70% compared to the control after dsRNA interference with the concentrations of 500 ng/μl, 1000 ng/μl, and 4000 ng/μl dsRNA, respectively. At the concentrations of 500 ng/μl, 1000 ng/μl, and 4000 ng/μl, tubulin genes were downregulated by 9%, 29%, and 51%, respectively, compared with the control. Statistical analysis showed that there were large, significant differences compared to the control group: *Dshsp90*, F(3, 8) = 137.5, *P* < 0.0001; *Dshsp70*, F(3, 8) = 623.7, *P* < 0.0001; and tubulin gene, F(3, 8) = 56.01, *P* < 0.0001. However, at 500 ng/μl concentration, Tukey’s test showed no significant difference (*P* > 0.05) relative to control (Fig. 8).

### Cold resistance test results

The concentration of 4000 ng/μl was used for the cold tolerance test. The female ticks of *D. silvarum* were incubated for 2 h at − 20 °C lethal temperature (LT50), and the number of deaths was calculated. The *Dshsp90* gene showed no significant difference in the number of fatalities (F(3, 8) = 3.467, *P* = 0.0709). However, the *Dshsp70* gene and tubulin gene dsRNA experimental groups' mortalities were significantly different from that of the control group (*Dshsp70*: F(3, 8) = 5.050, *P* = 0.0298, tubulin: F(3, 8) = 4.264, *P* = 0.0448). After the interference of *Dshsp90* gene, the average mortality in *D. silvarum* ticks increased from 40% in the blank control group to 70% in the dsRNA group. The interference of the Hsp70 gene led to increased mortality of *D. silvarum* ticks from 47.5% in the blank control group to 80% in the dsRNA group, whereas the average number of deaths rose from 37.5% in the blank control group to 70% in the dsRNA group after interference with the tubulin gene (Table 2).

**Table 2:** Analysis of cold tolerance of *Dermacentor silvarum* treated with RNAi

Gene	Mortality rate (mean ± SD)			
	Blank control	PBS	GFP	dsRNA
*HSP90*	40% ± 11%	45% ± 7%	60% ± 6%	70% ± 10%
*HSP70*	47.5% ± 7%	52.5% ± 8%	65% ± 14%	(80% ± 10%)*
Tubulin	37.5% ± 13%	47.5% ± 3%	57.5% ± 7%	(70% ± 10%)*

*PBS* Phosphate-buffered saline, *GFP* green fluorescent protein

^*^P < 0.05 indicates a significant difference

## Discussion

In the present study, two *D. silvarum Hsp* genes (*Dshsp70* and *Dshsp90*) were cloned and characterized, and their role under sub-lethal cold conditions after RNA interference was investigated. Heat shock (HS) genes, also known as stress genes, code for various proteins that form the earliest general stress defense system in organisms [55]. Hsp90 and Hsp70 display highly conserved amino acid sequences, and the results of the multiple alignments of the Hsps amino acid sequences in the present study underscore this high sequence identity. In *D. silvarum* Hsp90, three signature sequences and three other motifs were identified [48, 49]. Similarly to Hsp90s of other species, three major domains were identified in *D. silvarum* Hsp90, viz.: (i) an N-domain containing an ATP, radicicol, or geldanamycin binding pocket; (ii) a middle domain consisting of a catalytic site for the interaction of the ATP bound in the N-domain, which is also the site for client protein interactions; (iii) a C-domain which is essential for the dimerization of Hsp90 [50]. Additionally, it has been suggested that the well-conserved MEEVD motif at the C-terminal binds to numerous co-chaperones such as FKBPs, Hop/Sti1, and immunophilins Cyp40 [50], and this characteristic is an indication of the cytosolic identity of Hsp90 [48]. Hsp70s also possess similar domain structures that include a conserved ATPase domain, a peptide-binding domain, and a variable C-terminal region with a V/IEEVD-motif enabling the proteins to bind co-chaperones and other Hsps [56]. The co-chaperones Hop (aids protein folding activity) and the carboxy-terminus of Hsc70 interacting protein (CHIP), which exhibit ubiquitin ligase activity, contain a tetratricopeptide repeat (TPR) domain that binds to the well-conserved sequence EEVD-COOH in the C-terminal domains of Hsp90 and Hsp70 [30, 31, 57]. The sequences of both Hsps ending in EEVD motif at the C-terminal have significant functional implications. The Hop’s TPR2A domain specifically recognizes the C-terminal heptapeptide of Hsp70 while the TPR1 domain of Hop binds the C-terminal pentapeptide of Hsp90, which leads to the assembly of Hsp70–Hsp90 multichaperone complexes [26]. The *D. silvarum*-tubulin amino acid sequence in the present study consists of two signature sequences, which are MRE at the beginning of the N-terminal, and was corroborated by Kim and Denlinger [53], and DLAALEKDYEEVGIDSAEGAEDDGGEEF at the end of the C-terminal, which was confirmed by Nielsen et al. [54].

Although detailed structural information on Hsp70 and Hsp90 complexes with folding-competent substrates during different stress conditions is lacking, a study suggests that the multiple sites of interaction of Hsp70s result in conformational heterogeneity and fuzzy chaperone-substrate ensembles that enable them to circumvent kinetic traps in their conformational free energy landscape and efficiently fold to the native state [58]. Much of what is known about the mechanism of action of Hsp70 is obtained from the studies on *E. coli* Hsp70 Dnak. It is believed that Hsp70 chaperones across all species work via similar mechanisms because of the 50% identical nature of bacterial and mammalian Hsp70s with superimposable structures and similar enzymatic functions [59]. Substrate binding takes place in a hydrophobic pocket in the peptide-binding domain with an affinity that is a function of the nucleotide state of the ATPase domain. ATP hydrolysis causes structural changes in the ATPase domain, which induces conformational changes in the peptide-binding domain and C-terminal region that result in substrate trapping, implying that the function of Hsp70 requires the coordinated action of all three domains [60]. The low intrinsic capacity of Hsp70 to hydrolyse ATP and release ADP necessitates the transient interaction of Hsp70s with various cofactors, which include Hsp40s and tetratricopeptide repeat (TPR) co-chaperones [61, 62], and the simultaneous interaction with Hsp40 and substrate leads to a synergistic stimulation of ATPase. Sequentially, nucleotide exchange factors (NEFs) influence ATPase activity by facilitating return to the ATP-bound state [63, 64]. These various cofactors do not just influence the reaction cycle of Hsp70 but also provide functional variation and specificity among different Hsp70 proteins [65].

In response to rapid temperature changes, heat shock proteins induce different physiological, biochemical, and behavioral responses in arthropods [66]. Diurnal changes in temperature occur predominantly during the transition from day to night, but it happens slowly enough to allow arthropods to adapt by gradually adjusting their physiological state [67]. Rapid temperature changes of wide magnitude are seldom observed in nature except during extreme seasonal changes such as cold winter and hot summer months [68]. Temperature can significantly drop to negative degrees during cold winter months, and this has profound physiological and biochemical as well as behavioral implications in arthropods [69]. Thus, Hsps play an indispensable part in ensuring the survival and adaptation of arthropods during stress periods caused by cold and heat [70, 71]. Most arthropods enter into diapause to escape the extremes of temperature in the summer and the winter seasons, and Hsps have been found to have significant functions during diapause in a species-dependent manner [72, 73].

To understand the role of Hsps in rapid cold hardiness (RCH), an RNAi experiment was performed on hsp genes, and the results were tested by qPCR, after which the female *D. silvarum* ticks were exposed to sub-lethal low temperature. *DsHsp90* and *DsHsp70* were differentially expressed significantly with a fold change > 5. The cold resistance test showed that the tick group with *DsHsp70* gene knockdown had a significantly higher mortality rate, which implies an increased susceptibility to cold. This finding confirms the role of *Hsp70* not only in the development of rapid cold hardiness but also in cold stress recovery, and this was corroborated by the significant induction of *Hsp70* by cold with different temporal expression patterns in the insect *Bemisia tabaci* [74]. It was found that the cold survival of flesh flies is highly dependent on Hsps [13]. Similarly, *Hsp70* expression increased significantly in the blood lymphocytes after piglets were exposed at − 10 to − 4 °C for 0.5 h and subsequently returned to the pre-exposure level after 24 h [75]. It is noteworthy that protein unfolding as a result of cooling from room temperature to lower values is known as cold denaturation [76, 77]. Stress leads to the denaturation of nucleoprotein with the exposure and interaction of hydrophobic regions, causing aggregation. This swiftly triggers the movement of Hsp70 from the cytoplasm to the nucleus to bind with the hydrophobic regions to limit the degree of aggregation and to unfold the already aggregated proteins [75]. Thus, this process underscores the protective role of Hsp70 and how they influence the return of the unfolded proteins to their regular conformation. Additionally, *Hsp70* is expressed slightly differently to cold in *Drosophila melanogaster*, where it is upregulated after 1 h of recovery from cold exposure [78]. A possible explanation could be the role of *Hsp70* in the repair of cold injury in cells expressed as protein denaturation or misfolding [79]. Meanwhile, a particular strain of *Hsp70* gene was also induced in *Dr. melanogaster* during cold stress [80]. Generally, arthropods function and develop within a particular temperature range, and when they are exposed to the extremes of temperature, they will first be knocked down, then fall into a coma, and eventually die [81]. Another study found that, although both heat and cold-induced the upregulation of *Hsp70* genes, only two *Hsp70* strains (Hspa1a and Hspa8) were found to be upregulated during winter in both heat- and cold-adapted goats [23]. It emerged that *Hsp70* genes were expressed more highly in heat-adapted goats during winter and in cold-adapted goats during summer, suggesting that the pattern of *Hsp70* gene expression is species- and breed-specific, probably because of varying degrees of thermal tolerance and acclimatization to seasonal variations [23].

In contrast to *Hsp70* gene, the knockdown of *Hsp90* gene in the present study could not produce significant mortality in female *D. silvarum* after exposure to low temperature. Similarly, findings from another study indicated that *Hsp70* and *Hsp90* were inducible by rapid change in temperature, but *Hsp70* was more strongly induced than *Hsp90* [49]. In *B. tabaci*, the expression of *Hsp90*, contrary to *Hsp70*, was not affected during exposure to cold and recovery from cold hardening [74]. However, *Hsp90* played vital roles in the rapid cold hardiness of other insects, including *Locusta migratoria* [82] and *Chilo suppressalis* [51]. Owing to the similar basal level of *Hsp90* to *Hsp70*, which was more significant than *Hsp20* in *B. tabaci*, it was suggested that *Hsp90*’s primary role could be that of a housekeeping function [74], and it could also prevent the mis-aggregation of denatured proteins during temperature fluctuations [83]. Conversely, it appears that *Hsp90* was upregulated during heat stress in some species, as shown in the non-diapausing pupae of *Delia antiqua* [20]. These findings reflect significant variations on how different *Hsps* are expressed in low and high temperatures in other species at different phases. A possible explanation could be that species differences reflect differences in heat/cold tolerance mechanisms [84].

Tubulins are proteins that are often polymerized into microtubules, which are one of the major components of the cytoskeleton, and these microtubules are involved in various cellular processes such as mitosis, cell support, cell movement, and cell motility [35, 85]. In the present study, *D. silvarum* female ticks were exposed to a sub-lethal low temperature after the knockdown of the tubulin gene, and significant mortality was recorded compared to the blank control group. This could be attributed to the loss of the cells’ ability to adapt to low temperatures since the depolymerization and disassembly of microtubules take place at low temperatures, which putatively enhance cold tolerance [36]. The polymerization and depolymerization of microtubules in *Culex pipiens* were influenced by temperature and diapause, and this underscores the role that tubulins, as well as actin filaments, play in the cold response of arthropods [53, 86]. The depolymerization process of microtubules during low temperature is initiated by GTP hydrolysis [87] and facilitated by calcium ions as shown in the Antarctic insect *Belgica Antarctica*'s cold response mediated by calcium, which functions as the second messenger [88]. It is noteworthy that there is still a limited mechanistic understanding of the nature of cellular cryoinjury and mechanisms behind some animals having greater freeze tolerance than others. A study with *Chrymomyza costata* (malt fly) observed the non-lethal consequence of α-tubulin disruption upon freezing, which was unrepaired upon recovery of the insect larvae but could not conclude on the functional consequences for the cells [89]. The function and cold stability of microtubules are more evident in plants compared to those of animals owing to their higher developmental plasticity [90]. Due to their relative rigidity coupled with their innate nonlinear dynamics, plant microtubules have been proposed to be pre-adapted for a function as mechanosensors that decode stress-related signal signatures (including osmotic or cold stress) that is facilitated by phospholipase D [90]. Additionally, a very dynamic temperature-dependent binding of MAP6-F (a temperature sensor) to microtubules was observed in mouse fibroblast cells suggesting that MAP6 is a temperature sensor that adapts its conformation to maintain the cellular microtubule network in organisms exposed to low temperatures [91]. These findings portray low temperature as one of the factors that induce a fundamental change in tubulin development, with calcium playing the role of a signaling molecule to activate such changes.

## Conclusions

The findings of this study highlight the roles that *Dshsp70* and tubulin play in the adaptation of *D. silvarum* to low temperatures. *Dshsp70* gene expression can be utilized as a marker for the cold adaptation of different tick species. The information generated in this study can aid in the understanding of the survival and acclimatization of overwintering ticks.

## Supplementary Information

**Additional file 1:** **Figure S1.** Sequence data for Dshsp70, Dshsp90, and tubulin genes.

**Additional file 2:** **Figure S2.** Primer verification by PCR amplification of target genes (a: Dshsp90; b: Dshsp70; c: *Dermacentor silvarum* tubulin gene).

**Additional file 3:** **Figure S3.** Gene cloning after ligation of T7 promoter. (a) Dshsp 90 gene; (b) Dshsp 70 gene; (c) tubulin gene.

**Additional file 4:** **Table S1.** Gene-specific primers used in the present study.

## Data Availability

Data supporting the conclusions of this article are included within the article. The complete genome sequence of the heat shock proteins and tubulin of *D. silvarum* was deposited in the NCBI nucleotide database under the accession numbers MT646143 (Dshsp90), MT679220 (Dshsp70), and MT646144 (tubulin gene). Raw data are available from the corresponding author on reasonable request.

## Abbreviations

aa: Amino acids
nt: Nucleotide
Desil: *Dermacentor silvarum*
Emonu: *Empoasca onukii*
Ersin: *Eriocheir sinensis*
Gryfi: *Gryllus firmus*
Laost: *Laodelphax striatellus*
Parus: *Paratlanticus ussuriensis*
Potri: *Portunus trituberculatus*
Procl: *Procambarus clarkia*
Rhimi: *Rhipicephalus microplus*
Arave: *Araneus ventricosus*
Devar: *Dermacentor variabilis*
Dimoj: *Diguetia mojavea*
Disig: *Diguetia signata*
Hafla: *Haemaphysalis flava*
Kigua: *Kibramoa guapa*
Plete: *Plectreurys tecate*
Stemi: *Stegodyphus mimosarum*
Amvie: *Amphitetranychus viennensis*
Boter: *Bombus terrestris*
Brodo: *Bradysia odoriphaga*
Debre: *Demodex brevis*
Defol: *Demodex folliculorum*
Hasal: *Harpegnathos saltator*
Ixosc: *Ixodes scapularis*
Lipol: *Limulus polyphemus*

## References

[1] de la Fuente J, Estrada-Pena A, Venzal JM, Kocan KM, Sonenshine DE (2008). Overview: ticks as vectors of pathogens that cause disease in humans and animals. Front Biosci.

[2] Yu Z, Wang H, Wang T, Sun W, Yang X, Liu J (2015). Tick-borne pathogens and the vector potential of ticks in China. Parasit Vectors.

[3] Sonenshine DE, Roe RM (2013). Biology of ticks.

[4] Yu Z, Jia Q, Wang T, Dong N, Yang X, Wang H (2017). Gene expression profiling of the unfed nymphal *Dermacentor silvarum* (Acari: Ixodidae) in response to low temperature. Syst Appl Acarol.

[5] Randolph SE (2004). Tick ecology: processes and patterns behind the epidemiological risk posed by ixodid ticks as vectors. Parasitology.

[6] Oorebeek M, Kleindorfer S (2008). Climate or host availability: what determines the seasonal abundance of ticks?. Parasitol Res.

[7] Brunner JL, Killilea M, Ostfeld RS (2012). Overwintering survival of nymphal *Ixodes scapularis* (Acari: Ixodidae) under natural conditions. J Med Entomol.

[8] Lu Z, Broker M, Liang G (2008). Tick-borne encephalitis in mainland China. Vector Borne Zoonotic Dis.

[9] Tian ZC, Liu GY, Shen H, Xie JR, Luo J, Tian MY (2012). First report on the occurrence of *Rickettsia slovaca* and *Rickettsia raoultii* in *Dermacentor silvarum* in China. Parasit Vectors.

[10] Wen J, Jiao D, Wang JH, Yao DH, Liu ZX, Zhao G (2014). *Rickettsia raoultii*, the predominant Rickettsia found in *Dermacentor silvarum* ticks in China–Russia border areas. Exp Appl Acarol.

[11] Teng K, Jiang Z (1991). Acari: Ixodidae. Economic insect fauna of China.

[12] Yu Z, Zheng H, Yang X, Chen Z, Wang D, Hao M (2011). Seasonal abundance and activity of the tick *Dermacentor silvarum* in northern China. Med Vet Entomol.

[13] Rinehart JP, Li A, Yocum GD, Robich RM, Hayward SA, Denlinger DL (2007). Up-regulation of heat shock proteins is essential for cold survival during insect diapause. Proc Natl Acad Sci USA.

[14] Feder ME, Hofmann GE (1999). Heat-shock proteins, molecular chaperones, and the stress response: evolutionary and ecological physiology. Annu Rev Physiol.

[15] Muller P, Ruckova E, Halada P, Coates PJ, Hrstka R, Lane DP (2013). C-terminal phosphorylation of Hsp70 and Hsp90 regulates alternate binding to co-chaperones CHIP and HOP to determine cellular protein folding/degradation balances. Oncogene.

[16] Schopf FH, Biebl MM, Buchner J (2017). The HSP90 chaperone machinery. Nat Rev Mol Cell Biol.

[17] Genest O, Wickner S, Doyle SM (2019). Hsp90 and Hsp70 chaperones: collaborators in protein remodeling. J Biol Chem.

[18] Höhfeld J, Cyr DM, Patterson C (2001). From the cradle to the grave: molecular chaperones that may choose between folding and degradation. EMBO Rep.

[19] Radli M, Rüdiger SGD (2018). Dancing with the diva: Hsp90-client interactions. J Mol Biol.

[20] Chen B, Kayukawa T, Monteiro A, Ishikawa Y (2005). The expression of the HSP90 gene in response to winter and summer diapauses and thermal-stress in the onion maggot, *Delia**antiqua*. Insect Mol Biol.

[21] Benoit JB, Lopez-Martinez G, Phillips ZP, Patrick KR, Denlinger DL (2010). Heat shock proteins contribute to mosquito dehydration tolerance. J Insect Physiol.

[22] Tian Z, Liu G, Zhang L, Yin H, Wang H, Xie J (2011). Identification of the heat shock protein 70 (HLHsp70) in *Haemaphysalis longicornis*. Vet Parasitol.

[23] Banerjee D, Upadhyay RC, Chaudhary UB, Kumar R, Singh S, Ashutosh (2014). Seasonal variation in expression pattern of genes under HSP70 family in heat- and cold-adapted goats (Capra hircus). Cell Stress Chaperones.

[24] Doyle SM, Hoskins JR, Kravats AN, Heffner AL, Garikapati S, Wickner S (2019). Intermolecular interactions between Hsp90 and Hsp70. J Mol Biol.

[25] Johnson BD, Schumacher RJ, Ross ED, Toft DO (1998). Hop modulates Hsp70/Hsp90 interactions in protein folding. J Biol Chem.

[26] Scheufler C, Brinker A, Bourenkov G, Pegoraro S, Moroder L, Bartunik H (2000). Structure of TPR domain-peptide complexes: critical elements in the assembly of the Hsp70–Hsp90 multichaperone machine. Cell.

[27] Kirschke E, Goswami D, Southworth D, Griffin PR, Agard DA (2014). Glucocorticoid receptor function regulated by coordinated action of the Hsp90 and Hsp70 chaperone cycles. Cell.

[28] Bhattacharya K, Weidenauer L, Luengo TM, Pieters EC, Echeverría PC, Bernasconi L (2020). The Hsp70–Hsp90 co-chaperone Hop/Stip1 shifts the proteostatic balance from folding towards degradation. Nat Commun.

[29] Morishima Y, Kanelakis KC, Silverstein AM, Dittmar KD, Estrada L, Pratt WB (2000). The Hsp organizer protein hop enhances the rate of but is not essential for glucocorticoid receptor folding by the multiprotein Hsp90-based chaperone system. J Biol Chem.

[30] Liu FH, Wu SJ, Hu SM, Hsiao CD, Wang C (1999). Specific interaction of the 70-kDa heat shock cognate protein with the tetratricopeptide repeats. J Biol Chem.

[31] Ramsey AJ, Russell LC, Whitt SR, Chinkers M (2000). Overlapping sites of tetratricopeptide repeat protein binding and chaperone activity in heat shock protein 90. J Biol Chem.

[32] Lakhotia SC, Prasanth KV (2002). Tissue- and development-specific induction and turnover of hsp70 transcripts from loci 87A and 87C after heat shock and during recovery in *Drosophila melanogaster*. J Exp Biol.

[33] Mahroof R, Yan Zhu K, Neven L, Subramanyam B, Bai J (2005). Expression patterns of three heat shock protein 70 genes among developmental stages of the red flour beetle, *Tribolium castaneum* (Coleoptera: Tenebrionidae). Comp Biochem Physiol A Mol Integr Physiol.

[34] Li M, Lu WC, Feng HZ, He L (2009). Molecular characterization and expression of three heat shock protein70 genes from the carmine spider mite, *Tetranychus cinnabarinus* (Boisduval). Insect Mol Biol.

[35] McKean PG, Vaughan S, Gull K (2001). The extended tubulin superfamily. J Cell Sci.

[36] Pucciarelli S, Miceli C (2002). Characterization of the cold-adapted α-tubulin from the psychrophilic ciliate *Euplotes focardi*. Extremophiles.

[37] Nijhof AM, Taoufik A, de la Fuente J, Kocan KM, de Vries E, Jongejan F (2007). Gene silencing of the tick protective antigens, Bm86, Bm91 and subolesin, in the one-host tick *Boophilus microplus* by RNA interference. Int J Parasitol.

[38] Ramamoorthi N, Narasimhan S, Pal U, Bao F, Yang XF, Fish D (2005). The Lyme disease agent exploits a tick protein to infect the mammalian host. Nature.

[39] Sukumaran B, Narasimhan S, Anderson JF, DePonte K, Marcantonio N, Krishnan MN (2006). An *Ixodes scapularis* protein required for survival of *Anaplasma phagocytophilum* in tick salivary glands. J Exp Med.

[40] Pal U, Li X, Wang T, Montgomery RR, Ramamoorthi N, Desilva AM (2004). TROSPA, an *Ixodes scapularis* receptor for *Borrelia burgdorferi*. Cell.

[41] Liu J, Liu Z, Zhang Y, Yang X, Gao Z (2005). Biology of *Dermacentor silvarum* (Acari: Ixodidae) under laboratory conditions. Exp Appl Acarol.

[42] Yu Z, Pei T, Zhang M, Wang T, Jia Q, Yang X (2020). Differential transcriptome profiling of the diapause and cold-responsive genes in unfed adult *Dermacentor silvarum* Olenev (Acari: Ixodidae). Syst Appl Acarol.

[43] Bailey TL, Boden M, Buske FA, Frith M, Grant CE, Clementi L (2009). MEME SUITE: tools for motif discovery and searching. Nucleic Acids Res.

[44] Kocan KM, Blouin E, de la Fuente J (2011). RNA interference in ticks. J Vis Exp.

[45] Agwunobi DO, Zhang M, Shi X, Zhang S, Zhang M, Wang T (2021). DNA methyltransferases contribute to cold tolerance in ticks *Dermacentor silvarum* and *Haemaphysalis longicornis* (Acari: Ixodidae). Front Vet Sci.

[46] Livak KJ, Schmittgen TD (2001). Analysis of relative gene expression data using real-time quantitative PCR and the 2(-Delta Delta C(T)) method. Methods.

[47] Wang T, Yang X, Jia Q, Dong N, Wang H, Hu Y (2017). Cold tolerance and biochemical response of unfed *Dermacentor silvarum* ticks to low temperature. Ticks Tick Borne Dis.

[48] Gupta RS (1995). Phylogenetic analysis of the 90 kD heat shock family of protein sequences and an examination of the relationship among animals, plants, and fungi species. Mol Biol Evol.

[49] Zhang Q, Denlinger DL (2010). Molecular characterization of heat shock protein 90, 70 and 70 cognate cDNAs and their expression patterns during thermal stress and pupal diapause in the corn earworm. J Insect Physiol.

[50] Pearl LH, Prodromou C (2006). Structure and mechanism of the Hsp90 molecular chaperone machinery. Annu Rev Biochem.

[51] Sonoda S, Fukumoto K, Izumi Y, Yoshida H, Tsumuki H (2006). Cloning of heat shock protein genes (hsp90 and hsc70) and their expression during larval diapause and cold tolerance acquisition in the rice stem borer, *Chilo suppressalis Walker*. Arch Insect Biochem Physiol.

[52] Tungjitwitayakul J, Tatun N, Singtripop T, Sakurai S (2008). Characteristic expression of three heat shock-responsive genes during larval diapause in the bamboo borer *Omphisa fuscidentalis*. Zoolog Sci.

[53] Kim M, Denlinger DL (2009). Decrease in expression of beta-tubulin and microtubule abundance in flight muscles during diapause in adults of *Culex pipiens*. Insect Mol Biol.

[54] Nielsen MG, Gadagkar SR, Gutzwiller L (2010). Tubulin evolution in insects: gene duplication and subfunctionalization provide specialized isoforms in a functionally constrained gene family. BMC Evol Biol.

[55] Garbuz DG (2017). Regulation of heat shock gene expression in response to stress. Mol Biol.

[56] Daugaard M, Rohde M, Jäättelä M (2007). The heat shock protein 70 family: Highly homologous proteins with overlapping and distinct functions. FEBS Lett.

[57] Russell LC, Whitt SR, Chen MS, Chinkers M (1999). Identification of conserved residues required for the binding of a tetratricopeptide repeat domain to heat shock protein 90. J Biol Chem.

[58] Rosenzweig R, Sekhar A, Nagesh J, Kay LE (2017). Promiscuous binding by Hsp70 results in conformational heterogeneity and fuzzy chaperone-substrate ensembles. eLife.

[59] Sharma D, Masison DC (2009). Hsp70 structure, function, regulation and influence on yeast prions. Protein Pept Lett.

[60] Han W, Christen P (2003). Interdomain communication in the molecular chaperone DnaK. Biochem J.

[61] Lu Z, Cyr DM (1998). Protein folding activity of Hsp70 is modified differentially by the hsp40 co-chaperones Sis1 and Ydj1. J Biol Chem.

[62] Wegele H, Haslbeck M, Reinstein J, Buchner J (2003). Sti1 is a novel activator of the Ssa proteins. J Biol Chem.

[63] Kabani M, McLellan C, Raynes DA, Guerriero V, Brodsky JL (2002). HspBP1, a homologue of the yeast Fes1 and Sls1 proteins, is an Hsc70 nucleotide exchange factor. FEBS Lett.

[64] Dragovic Z, Broadley SA, Shomura Y, Bracher A, Hartl FU (2006). Molecular chaperones of the Hsp110 family act as nucleotide exchange factors of Hsp70s. EMBO J.

[65] Young JC, Agashe VR, Siegers K, Hartl FU (2004). Pathways of chaperone-mediated protein folding in the cytosol. Nat Rev Mol Cell Biol.

[66] Sorensen JG, Nielsen MM, Kruhoffer M, Justesen J, Loeschcke V (2005). Full genome gene expression analysis of the heat stress response in *Drosophila melanogaster*. Cell Stress Chaperones.

[67] Denlinger DL, Lee RE (2010). Low temperature biology of insects.

[68] Benoit JB, Lopez-Martinez G, Patrick KR, Phillips ZP, Krause TB, Denlinger DL (2011). Drinking a hot blood meal elicits a protective heat shock response in mosquitoes. Proc Natl Acad Sci USA.

[69] Michaud MR, Denlinger DL (2004). Molecular modalities of insect cold survival: current understanding and future trends. Int Congr Ser.

[70] Kang L, Chen B, Wei JN, Liu TX (2009). Roles of thermal adaptation and chemical ecology in *Liriomyza* distribution and control. Annu Rev Entomol.

[71] Hu JT, Chen B, Li ZH (2014). Thermal plasticity is related to the hardening response of heat shock protein expression in two Bactrocera fruit flies. J Insect Physiol.

[72] MacRae TH (2010). Gene expression, metabolic regulation and stress tolerance during diapause. Cell Mol Life Sci.

[73] Cheng W, Li D, Wang Y, Liu Y, Zhu-Salzman K (2016). Cloning of heat shock protein genes (hsp70, hsc70 and hsp90) and their expression in response to larval diapause and thermal stress in the wheat blossom midge, *Sitodiplosis**mosellana*. J Insect Physiol.

[74] Wang H, Lei Z, Li X, Oetting RD (2011). Rapid cold hardening and expression of heat shock protein genes in the B-biotype *Bemisia tabaci*. Environ Entomol.

[75] Ji H, Wu Y, Guo J, Wang J, Li S, Yang H (2012). Effects of cold stress on expression of Hsp70 gene in blood lymphocyte of piglets. J Anim Vet Adv.

[76] Privalov PL (1990). Cold denaturation of proteins. Crit Rev Biochem Mol Biol.

[77] Sanfelice D, Temussi PA (2016). Cold denaturation as a tool to measure protein stability. Biophys Chem.

[78] Sinclair BJ, Gibbs AG, Roberts SP (2007). Gene transcription during exposure to, and recovery from, cold and desiccation stress in *Drosophila melanogaster*. Insect Mol Biol.

[79] Stetina T, Kostal V, Korbelova J (2015). The role of inducible Hsp70, and other Heat shock proteins, in adaptive complex of cold tolerance of the fruit fly (*Drosophila melanogaster*). PLoS ONE.

[80] Colinet H, Lee SF, Hoffmann A (2010). Temporal expression of heat shock genes during cold stress and recovery from chill coma in adult *Drosophila melanogaster*. FEBS J.

[81] Chown SL, Nicolson S (2004). Insect physiological ecology: mechanisms and patterns.

[82] Wang XH, Kang L (2005). Differences in egg thermotolerance between tropical and temperate populations of the migratory locust *Locusta migratoria* (Orthoptera: Acridiidae). J Insect Physiol.

[83] Sonna LA, Fujita J, Gaffin SL, Lilly CM (2002). Invited review: effects of heat and cold stress on mammalian gene expression. J Appl Physiol.

[84] Goto SG, Kimura MT (2004). Heat-shock-responsive genes are not involved in the adult diapause of *Drosophila triauraria*. Gene.

[85] Nogales E (2000). Structural insights into microtubule function. Annu Rev Biochem.

[86] Kim M, Robich RM, Rinehart JP, Denlinger DL (2006). Upregulation of two actin genes and redistribution of actin during diapause and cold stress in the northern house mosquito *Culex**pipiens*. J Insect Physiol.

[87] Erickson HP, O'Brien ET (1992). Microtubule dynamic instability and GTP hydrolysis. Annu Rev Biophys Biomol Struct.

[88] Teets NM, Elnitsky MA, Benoit JB, Lopez-Martinez G, Denlinger DL, Lee RE (2008). Rapid cold-hardening in larvae of the Antarctic midge *Belgica antarctica*: cellular cold-sensing and a role for calcium. Am J Physiol Regul Integr Comp Physiol.

[89] Des Marteaux LE, Štětina T, Koštál V (2018). Insect fat body cell morphology and response to cold stress is modulated by acclimation. J Exp Biol.

[90] Nick P (2013). Microtubules, signalling and abiotic stress. Plant J.

[91] Delphin C, Bouvier D, Seggio M, Couriol E, Saoudi Y, Denarier E (2012). MAP6-F is a temperature sensor that directly binds to and protects microtubules from cold-induced depolymerization. J Biol Chem.

