# Supplementary figures and images for: Functional implication of heat shock protein 70/90 and tubulin in cold stress of Dermacentor silvarum
Source: Parasit Vectors. 2021 Oct 19;14:542. doi: 10.1186/s13071-021-05056-y (PMC8527796; doi:10.1186/s13071-021-05056-y)

### Hsp70

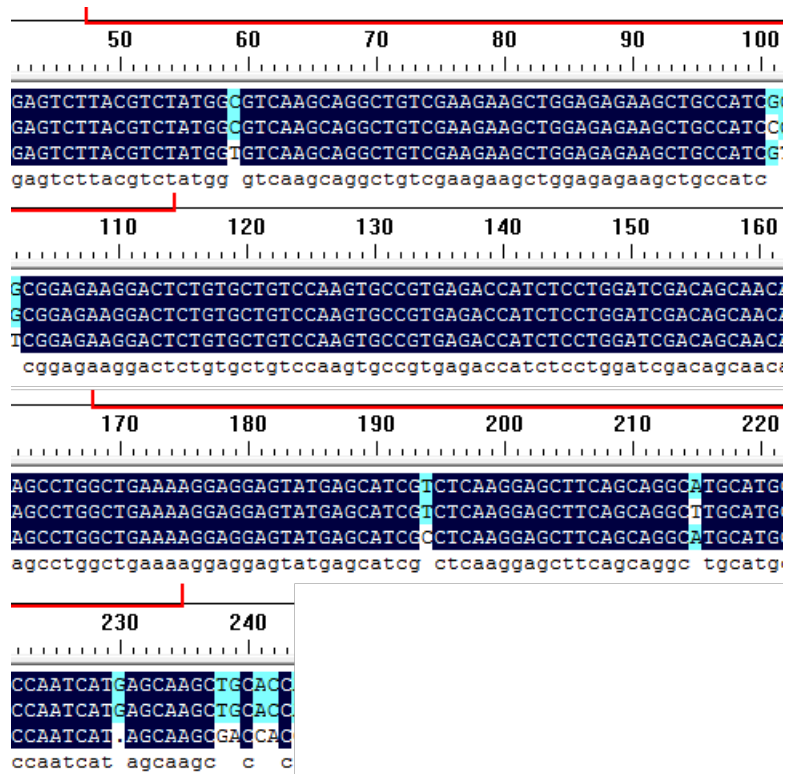

### Hsp90

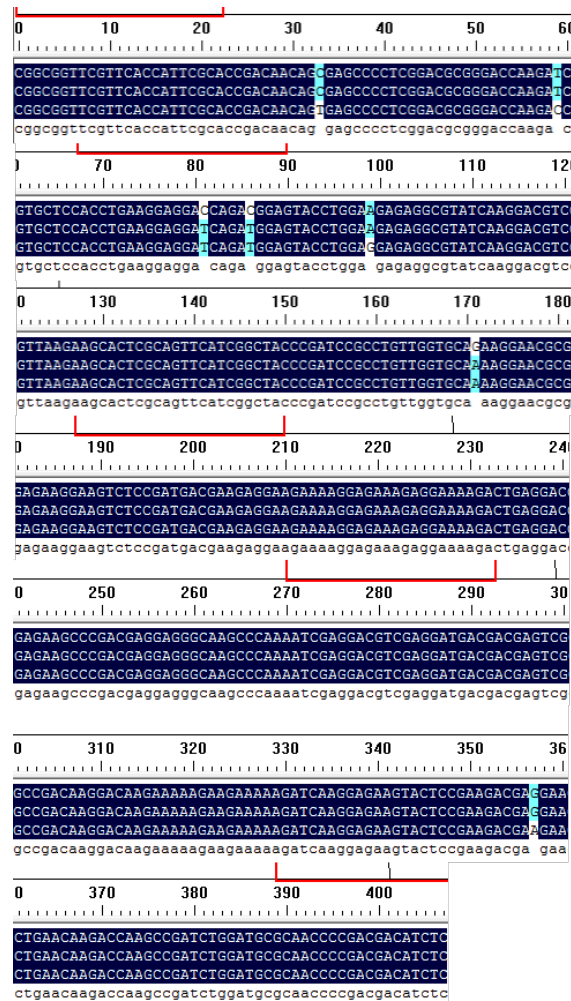

### Tubulin gene

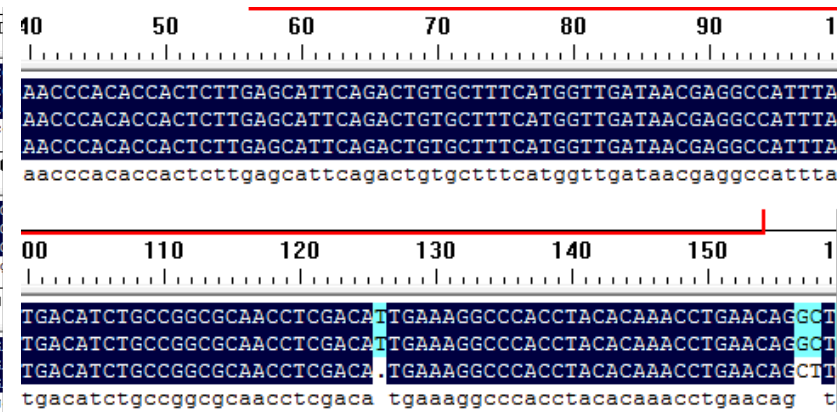

Additional file 1: Figure S1. Sequence data for *Hsp70*, *Hsp90*, and tubulin genes

Supplement: Supplementary file 1 — Additional file 1: Figure S1. Sequence data for Dshsp70, Dshsp90, and tubulin genes. [file 13071_2021_5056_MOESM1_ESM.pdf]
