# Supplementary material for: Functional implication of heat shock protein 70/90 and tubulin in cold stress of Dermacentor silvarum
Source: Parasit Vectors. 2021 Oct 19;14:542. doi: 10.1186/s13071-021-05056-y (PMC8527796; doi:10.1186/s13071-021-05056-y)
Supplement: Supplementary file 2 — Additional file 2: Figure S2. Primer verification by PCR amplification of target genes (a: Dshsp90; b: Dshsp70; c: Dermacentor silvarum tubulin gene). [file 13071_2021_5056_MOESM2_ESM.pdf]

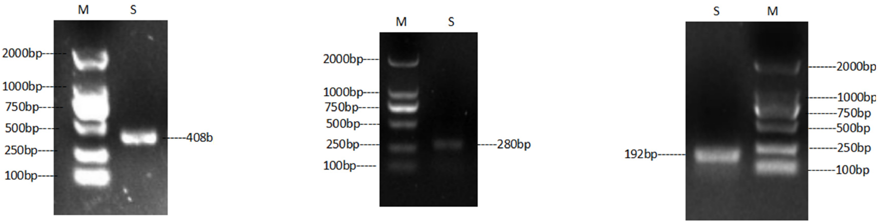

Hsp90 gene **Hsp70 gene** **Tubulin gene**

Additional file 2: Figure S2. Primer verification by PCR amplification of target genes
