# Supplementary material for: Functional implication of heat shock protein 70/90 and tubulin in cold stress of Dermacentor silvarum
Source: Parasit Vectors. 2021 Oct 19;14:542. doi: 10.1186/s13071-021-05056-y (PMC8527796; doi:10.1186/s13071-021-05056-y)
Supplement: Supplementary file 3 — Additional file 3: Figure S3. Gene cloning after ligation of T7 promoter. (a) Dshsp 90 gene; (b) Dshsp 70 gene; (c) tubulin gene. [file 13071_2021_5056_MOESM3_ESM.pdf]

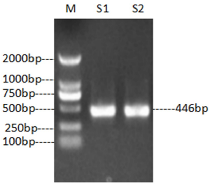

Hsp90 gene

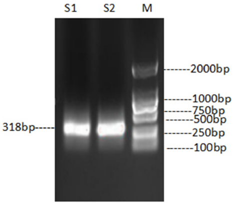

Hsp70 gene

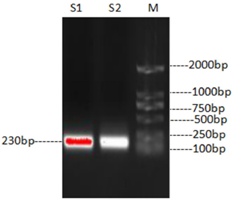

Tubulin gene

Additional file 3: Figure S3. Gene cloning after ligation of T7 promoter
