# Supplementary material for: Functional implication of heat shock protein 70/90 and tubulin in cold stress of Dermacentor silvarum
Source: Parasit Vectors. 2021 Oct 19;14:542. doi: 10.1186/s13071-021-05056-y (PMC8527796; doi:10.1186/s13071-021-05056-y)
Supplement: Supplementary file 4 — Additional file 4: Table S1. Gene-specific primers used in the present study. [file 13071_2021_5056_MOESM4_ESM.docx]

**Additional file 4: Table S1** Gene-specific primers used in the present study

| **Gene** | **Primer sequence [5ʹ˗3ʹ]** | **Purpose** |
| --- | --- | --- |
| Hsp90 | F: CGGCGGTTCGTTCACCATTC | Real-time PCR |
|  | R: GAGATGTCGTCGGGGTTGCG | Real-time PCR |
| Hsp70 | F: ACGATGCACAGCGCGAGAGG | Real-time PCR |
|  | R: TGGTGTGCTGCTGACCTGTG | Real-time PCR |
| Tubulin | F: CTCTACAGCAGTCGTGGAACC | Real-time PCR |
|  | R: GAGGCAGTGATTGAGGAGACGAT | Real-time PCR |
| B-actin | F: CGTTCCTGGGTATGGAATCG | Real-time PCR |
|  | R: TCCACGTCGCACTTCATGAT | Real-time PCR |
| GFP | F1: GACGTAAACGGCCACAAGT | Real-time PCR |
|  | R1: TAATACGACTCACTATAGGGCTTCTCGTTGGGGTCTTT | Real-time PCR |
| GFP | F2: TAATACGACTCACTATAGGGACGTAAACGGCCACAAGT | Real-time PCR |
|  | R2: GCTTCTCGTTGGGGTCTTT | Real-time PCR |
| Hsp90 | F: TAATACGACTCACTATAGGCGGCGGTTCGTTCACCATTC | dsRNA synthesis |
|  | R: TAATACGACTCACTATAGGGAGATGTCGTCGGGGTTGCG | dsRNA synthesis |
| Hsp70 | F: TAATACGACTCACTATAGGACGATGCACAGCGCGAGAGG | dsRNA synthesis |
|  | R: TAATACGACTCACTATAGGTGGTGTGCTGCTGACCTGTG | dsRNA synthesis |
| Tubulin | F: TAATACGACTCACTATAGGCTCTACAGCAGTCGTGGAACC | dsRNA synthesis |
|  | R: TAATACGACTCACTATAGGGAGGCAGTGATTGAGGAGACGAT | dsRNA synthesis |
